# Some Conditions Which May Simulate Appendicitis

**Published:** 1908-02-29

**Authors:** 


### Points in Surgery.

### SOME CONDITIONS WHICH MAY SIMULATE APPENDICITIS.

In the last few years many aspects of surgery have undergone a remarkable change; and the modern treatment of appendicitis is not the least important result of this circumstance. It is not so long ago since all cases of appendicitis were put to bed and watched, irrespective of the gravity of the disorder in any particular case, with the result that the mortality from this disease was appallingly high. If any operation was performed at all it was delayed until the patient was *in articulo mortis*, so that the apparent results of surgical interference were hardly, if at all, better than those achieved by leaving the patient alone. Sir Frederick Treves was one of the first surgeons who preached and practised the doctrine that if good was to be done by surgical treatment, operation should not be indefinitely postponed; and he laid it down as a law that if there was no improvement in the patient's condition in five days from the onset of the attack, the time had come for operation. That was fifteen years ago or more. Since then the period of waiting has been still further reduced, so that, in many hospitals at any rate, operation is immediately performed if there are definite signs of abscess or of peritonitis, and even if these are absent, the appendix is generally removed if the local signs do not begin to abate in twenty-four hours at the most. The writer is not concerned in proving here that such treatment has reduced the mortality. Statistics prove it conclusively, and every logically-minded man must be convinced by the figures which have been brought forward to show this. Early operation is then the best possible treatment for the great majority of cases of appendicitis; for all cases, in fact, except possibly those in which the attack is definitely subsiding; and here the only object in waiting is that the appendix may be subsequently removed in the quiescent stage, for it has been definitely shown that the presence of an appendix which has once been inflamed is a danger to its possessor, because it may at any moment cause a second attack, which is often far more acute than the original one.

But there is a definite risk attending the immediate removal of the appendix at a very early stage in all cases of apparent appendicitis; and that is that in spite of the greatest possible care in making the diagnosis certain, the abdomen may be needlessly opened for some quite different condition. Those who are acquainted with the protean nature of this affection will appreciate the difficulty.

It is always, for instance, difficult to be sure of the diagnosis in the case of young women, because the various forms of pelvic inflammation to which they are subject may simulate appendicitis very closely. A young girl who comes up complaining of sudden pain in the right side of the hypogastrium and is found to have a palpable tumour in the right iliac fossa, may have appendicitis with or without abscess (in the latter case the mass is due simply to matted intestines) or she may have an inflammatory condition of the right Fallopian tube or ovary. Are there any points which throw light on the differential diagnosis? In the first place the attack is more acute in onset in appendicitis and is usually accompanied by vomiting; and in nearly all cases it follows on a period of constipation. In perimetritis, however acute, vomiting is not a constant phenomenon. The menstrual history of such a patient should be inquired into with the minutest care, and any irregularity is suggestive of a pelvic origin, as is also a history of leucorrhœa. Examination of the abdomen may give little information, except that if there be a mass in both iliac fossæ the case is more likely to be one of perimetritis. Examination *per vaginam* is of more assistance, for it may be possible to feel an inflamed tube, which makes the diagnosis of perimetritis more or less certain, and, further, the presence or absence of a vaginal discharge can be established. If leucorrhœa is present the case is probably one of uterine origin. No reliance can be placed upon the temperature as a means of diagnosis.

If a thorough examination does not entirely clear up the diagnosis, it is permissible to watch the patient for a short time, but only if the conditions allow of immediate operation in the event of the patient's condition becoming worse. If the case is one of perimetritis the symptoms will subside. But it should be remembered that it is less harmful to open the abdomen for perimetritis than it is to stand by while an inflamed appendix is setting up localised or general peritonitis. For though it is true that in the former case the patient may have been unnecessarily subjected to laparotomy, in the latter she may unnecessarily lose her life.

To show that even with the greatest care mistakes may occur, the following cases may be briefly mentioned. The first was a girl who had sudden pain in the right iliac fossa, and a palpable mass. She had no vomiting, but a history of leucorrhœa. The case was taken to be one of perimetritis, and expectant treatment was adopted. The symptoms subsided, but the mass was still there after some weeks of this, and the abdomen was opened on the supposition that the tumour was a residual pyosalpinx. At the operation it was found that the mass was due to intestines matted round a recently inflamed appendix. The other case was the exact counterpart. The appearances all pointed to an attack of appendicitis. The patient had sudden severe pain in the right iliac fossa, with vomiting. There was no menstrual irregularity and no leucorrhœa. Yet, when the abdomen was opened, the cause of the symptoms was found to be an acutely inflamed tube with retained pus.

In some cases the appendix and tube are both found to be inflamed. In these the infection is generally primarily of pelvic origin, the appendix being involved later. This is only what is to be expected if one remembers the bacteriology of the two conditions. For it is more likely that the staphylococcus and gonococcus, which are responsible for pelvic inflammation, should infect the peritoneum, than that the bacillus coli should involve the genital apparatus. These cases cannot therefore be classed as genuine cases of appendicitis: they differ from the ordinary form of the disease clinically as well as bacteriologically. For even though the appendix is involved, cases of pelvic inflammation of uterine origin can be put to bed and watched; the only treatment that need be adopted is absolute rest, combined with hot vaginal douches and the application of fomentations to the hypogastrium. These simple measures are usually all that is required; and it may be confidently predicted that the attack will quiet down if it is of uterine origin. In most cases the recovery will be complete; in quite a small number a persistent tumour will remain after the acute symptoms have disappeared, and if so a laparotomy may have to be performed eventually, when it will be found that the tumour consists of a residual abscess in or round the Fallopian tube. Unlike appendicitis due to the bacillus coli, it is an event of extreme rarity for abscesses of uterine origin to give rise to a general peritonitis. The peritoneum seems to have an infinitely greater power of resistance to the gonococcus and the staphylococcus than to the bacillus coli.

Another condition which may be most misleading to the surgeon is the presence of an appendix abscess occupying an unusual situation in the abdominal cavity. The following case is a good example of this difficulty. A small child complained of pain in the left side of the hypogastrium, accompanied by vomiting. Her temperature was 102, and her pulse 120. Expectant treatment was adopted, and she apparently recovered completely in a few days. But in a fortnight all the symptoms returned with greater severity than before. She was in such pain that a satisfactory examination of the abdomen was impossible. She was therefore anæsthetised with chloroform, and it was then found that there was a definite mass in the left iliac fossa. Its lower edge extended down over the brim of the pelvis, and it was apparently fixed. It was dull on percussion. In view of her general symptoms it was concluded that the mass was an abscess, but what its exact nature was it was impossible to say. The abdomen was opened by a vertical incision through the left rectus abdominis muscle, and on separating the coils of intestines, which were adherent to each other, a large abscess was evacuated. It appeared to be bounded by small gut on one side and descending colon on the other. The pus had the odour which is only associated with abscesses due to the bacillus coli. On investigating the abscess cavity it was found to be connected with the appendix, whose tip communicated with the cavity at its right boundary. It was not considered feasible to remove the appendix. The patient made an uneventful recovery. The case is quoted as showing how anomalous may be the tumour caused by what is really an appendix abscess.

Where there is no tumour the case may be more difficult still, because often the only sign which the surgeon can rely on in making his diagnosis is the localisation of the pain and tenderness. Now it is a matter of common knowledge that even in definite cases of appendicitis the pain is referred not always to the right iliac fossa, but often to the neighbourhood of the umbilicus; and this is the case with several other intra-abdominal conditions, particularly inflammatory conditions of the gall-bladder. In chronic cholecystitis, secondary say, to a gall-stone, the gall-bladder may be enlarged and adherent to the posterior surface of the abdominal wall, and the only symptom of which the patient complains may be pain round the umbilicus or to the right of it. The typical symptoms of disease of the gall-bladder may be entirely absent, and in this case the diagnosis may be astonishingly difficult. Yet another condition which may mislead one in the same way is a movable kidney on the right side. The symptoms to which this condition gives rise appear to differ in every case, and it is for this reason that the cause is often overlooked, and even if the patient is examined under an anæsthetic the true state of affairs may not be discovered; and for this reason that a movable kidney can only be palpated when the patient inspires deeply, which he does not do under the anæsthetic. Those who are acquainted with the practical aspect of abdominal surgery will not need to be told that there are cases in which it is not humanly possible to make a certain diagnosis where the symptoms point to a lesion in the right side of the abdominal cavity. It is in these cases that a vertical incision through or to the outer side of the right rectus muscle is especially serviceable. Through it the appendix region can be explored with moderate ease, and the organ removed if necessary; while, if it is not at fault, the incision can be enlarged upwards and the abdomen systematically explored until the cause of the trouble is located.

Tuberculous peritonitis may simulate appendicitis more or less closely, especially in that variety of the disease in which adjacent coils of intestine are matted together by adhesions, and a tumour is thus formed in the right iliac fossa. The points on which to rely are the aspect of the patient, and the sub-acute onset in the case of tuberculous peritonitis. The age may also be some guide, because tuberculous peritonitis is commoner in children; and the younger the child the greater the probability that the symptoms are due to this condition. The diagnosis may be made more certain by testing the opsonic index of the patient to the tubercle bacillus. Fortunately, however, laparotomy is equally beneficial in either case. For merely opening the abdomen seems to determine a cure in many cases of tuberculous peritonitis. The exact relation of cause and effect is not easy to explain.

Finally, certain conditions which lie within the domain of the physician may simulate appendicitis. The commonest of these is a lobar pneumonia of the base of the right lung, especially when the consolidation comes to the surface on its diaphragmatic aspect, so that the physical signs are obscure. Such a pneumonia may cause a rigidity of the abdomen all down the right side, owing to referred pain, and the appendix may be considered to be at fault. But the general flushed aspect of the patient, the sudden high rise of temperature, and the rapidity of the patient's breathing should be quite sufficient to direct attention to the cause of the symptoms.

